# FNDC3B promotes epithelial-mesenchymal transition in tongue squamous cell carcinoma cells in a hypoxic microenvironment

**DOI:** 10.3892/or.2021.8067

**Published:** 2021-04-27

**Authors:** Zhaoming Zhong, Hongxing Zhang, Min Hong, Chuanzheng Sun, Yuanyuan Xu, Xiao Chen, Change Gao, Minjie He, Weiqing Liu, Jin Liang

Oncol Rep 39: 1853-1859, 2018; DOI: 10.3892/or.2018.6231

After the publication of the article, and also the publication of a Corrigendum (see doi: 10.3892/or.2020.7744), there are further errors in the published paper that the authors wish to correct in a subsequent corrigendum. In the printed version of Fig. 5, the “NC” images were mistakenly presented in the data panels showing the results of the TCA8113 and TSCCA invasion assay experiments. Furthermore, in Fig. 4A and 6A, the β-actin control bands were erroneously selected for these figures.

The corrected versions of Figs. 4, 5 and 6 are shown opposite and on the next page, incorporating the correct data for Figs. 4A, 5 and 6A. These further corrections do not affect the results and conclusions of this work. The authors all agree to this Corrigendum, and are grateful to the Editor of *Oncology Reports* for allowing them to have the opportunity to correct these additional errors. Lastly, the authors apologize to the readership for any inconvenience these errors may have caused.

## Figures and Tables

**Figure 4.**
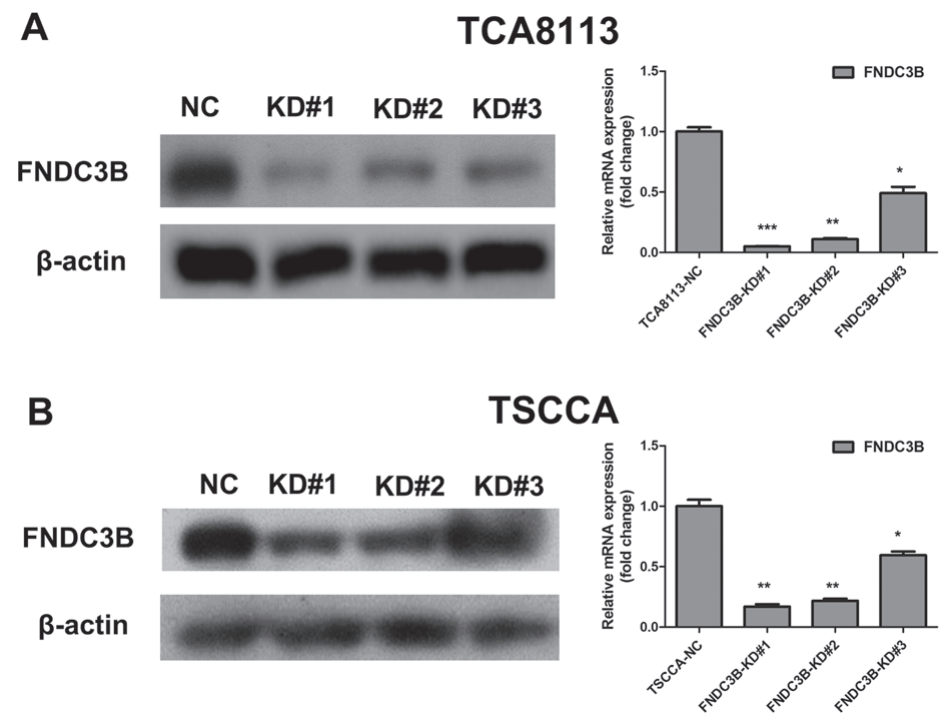
After knockdown of FNDC3B, the protein and mRNA levels of FNDC3B in (A) TCA8113 and (B) TSCCA cells were significantly decreased, as measured by western blotting and real-time PCR, respectively. *P<0.05, **P<0.01, ***P<0.001 compared with the NC-transfected cells.

**Figure 5.**
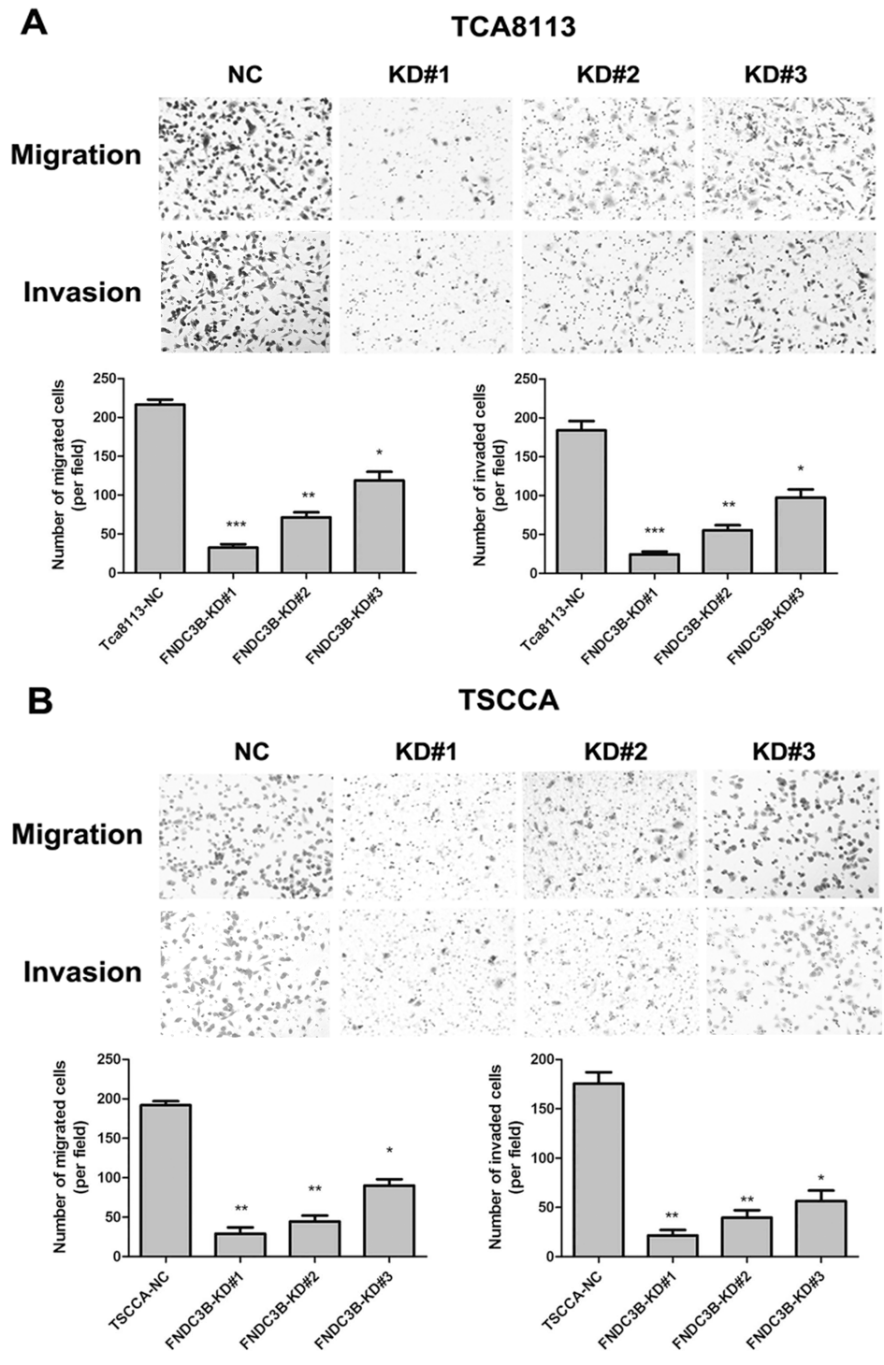
After knockdown of FNDC3B, the invasive and migratory abilities of (A) TCA8113 and (B) TSCCA cells were significantly decreased, as assessed by Transwell assay. *P<0.05, **P<0.01, ***P<0.001 compared to the NC-transfected cells. Photomicrographs are at ×100 magnification.

**Figure 6.**
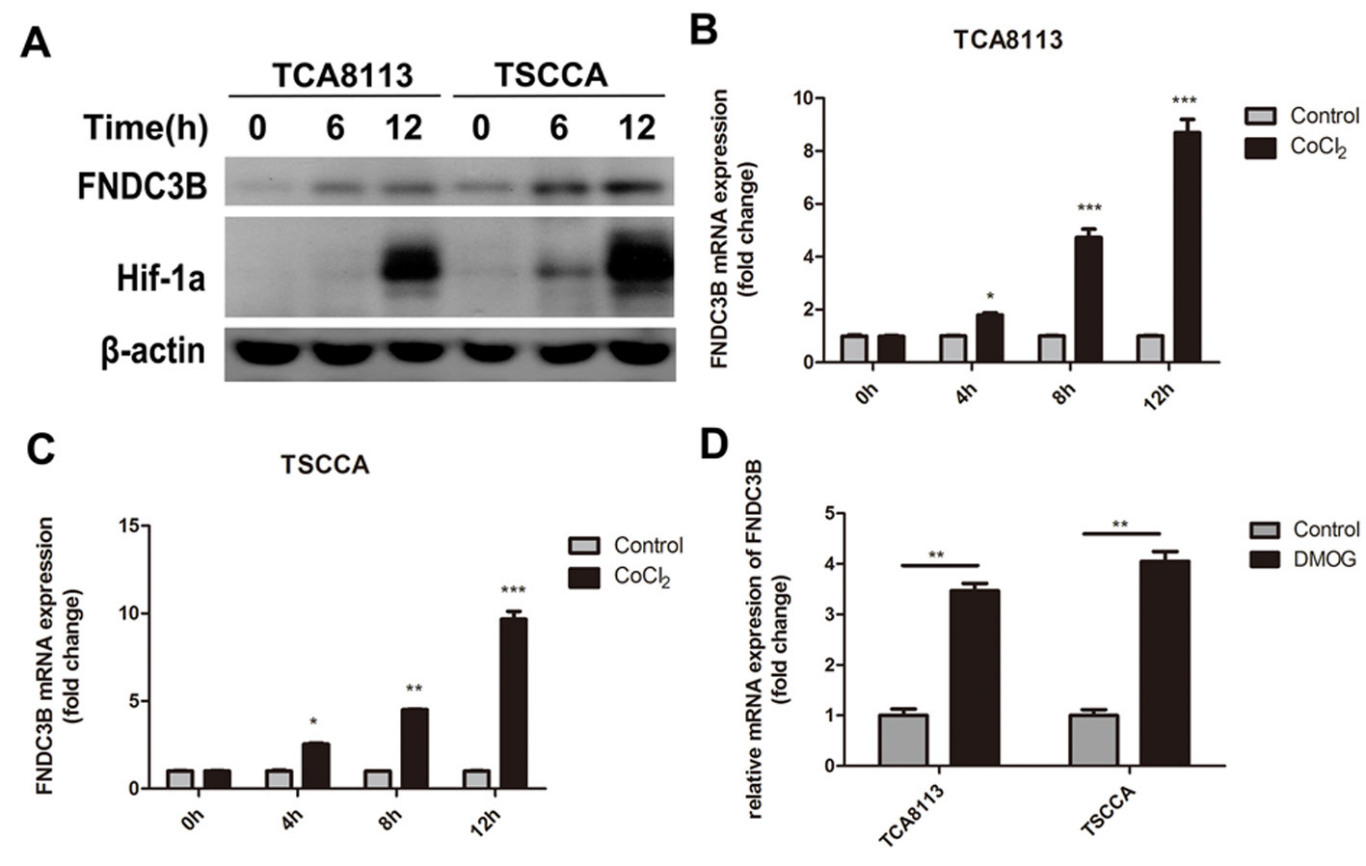
CoCl2 induces FNDC3B expression. (A) Protein expression and relative mRNA levels of FNDC3B in (B) TCA8113 and (C) TSCCA cells induced by 0.1 mM CoCl_2_ at the indicated times, as measured by real-time PCR and western blotting. (D) Protein expression of FNDC3B in TCA8113 cells induced by 1 mM DMOG. *P<0.05, **P<0.01, ***P<0.001 compared to the relevant control.

